# Laboratory model test study of the hydrological effect on granite residual soil slopes considering different vegetation types

**DOI:** 10.1038/s41598-021-94276-4

**Published:** 2021-07-19

**Authors:** Jie Chen, Xue-wen Lei, Han-lin Zhang, Zhi Lin, Hui Wang, Wei Hu

**Affiliations:** 1 Wuhan University of Science and Technology, Wuhan, 430065 China; 2 Zhongkai University of Agriculture and Engineering, Guangzhou, 510225 China; 3 Hunan Province Key Laboratory of Geotechnical Engineering Stability Control and Health Monitoring, Xiangtan, 411201 China

**Keywords:** Hydrology, Natural hazards

## Abstract

The problems caused by the interaction between slopes and hydrologic environment in traffic civil engineering are very serious in the granite residual soil area of China, especially in Guangdong Province. Against the background of two heavy rainfall events occurring during a short period due to a typhoon making landfall twice or even two typhoons consecutively making landfall, laboratory model tests were carried out on the hydrological effects of the granite residual soil slope considering three vegetation types under artificial rainfall. The variation in slope surface runoff, soil moisture content and rain seepage over time was recorded during the tests. The results indicate that surface vegetation first effectively reduces the splash erosion impact of rainwater on slopes and then influences the slope hydrological effect through rainwater forms adjustment. (1) The exposed slope has weak resistance to two consecutive heavy rains, the degree of slope scouring and soil erosion damage will increase greatly during the second rainfall. (2) The multiple hindrances of the stem leaf of *Zoysia japonica* plays a leading role in regulating the hydrological effect of slope, the root system has little effect on the permeability and water storage capacity of slope soil, but improves the erosion resistance of it. (3) Both the stem leaf and root system of *Nephrolepis cordifolia* have important roles on the hydrological effect. The stem leaf can stabilize the infiltration of rainwater, and successfully inhibit the surface runoff under continuous secondary heavy rainfall. The root system significantly enhances the water storage capacity of the slope, and greatly increases the permeability of the slope soil in the second rainfall, which is totally different from that of the exposed and *Zoysia japonica* slopes. (4) Zoysia is a suitable vegetation species in terms of slope protection because of its comprehensive slope protection effect. *Nephrolepis cordifolia* should be cautiously planted as slope protection vegetation. Only on slopes with no stability issues should *Nephrolepis cordifolia* be considered to preserve soil and water.

Protecting, restoring and promoting the sustainable use of terrestrial ecosystems, combating desertification, and halting and reversing soil degradation are among the 17 global development goals set by the United Nations by 2030. However, the development of human economy and society is difficult to avoid the destruction of ecological environment, especially in the field of traffic engineering construction^1,2^. Existing studies have indicated that roads, especially highways, inevitably impact the surrounding ecological environment. Their effects include landscape fragmentation, alteration of the topography and watershed hydrological processes, and water quality change^3–5^. Moreover, vegetation is readily damaged, and a large number of exposed slopes are typically generated in road construction. Under the effect of heavy rainfall, serious erosion phenomena often occurs in the slope areas on both sides of roads^6–8^. Specifically, high and steep cutting slopes are intensely scoured by the incoming water originating from upward slope areas, and the areas along roads exhibit a high incidence of geological disasters such as collapses and landslides. Due to the unconsolidated soil quality and poor erosion resistance of filled slopes, rill or gully erosion easily occurs under the influence of rainfall and road catchment water runoff^9^. When gully erosion develops on a slope surface, the number of erosion gullies sharply increases^10^. In addition, road subgrades with a high density and low infiltration are an important source of excess infiltration and surface runoff in mountainous areas^11^. Moreover, the occurrence of road surfaces and channels also accelerates the water collection process and runoff velocity^12^, which generates adverse impacts on the ecological environment and even threatens the stability of road slopes^13,14,15,16,17^.

Granite is widely distributed in southeastern China, especially in Guangdong and Fujian provinces, where the exposed area accounts for 30–40% of the total area. Under the effect of long-term geological and climatic conditions, the granite surface is generally covered with a thick layer of weathered eluvial soil, namely, granite residual soil, which is a characteristic regional soil and one of the main soils encountered in engineering construction in the coastal areas of the Yuemin region. In recent years, with the development of the Guangdong-Hong Kong-Macao Greater Bay Area, infrastructure construction in Guangzhou and surrounding areas has ushered in a new round of development opportunities. However, the region is dominated by a subtropical monsoon climate, with an annual average rainfall higher than 2000 mm. Moreover, precipitation is concentrated, continuous heavy rainfall occasionally occurs due to a given typhoon making landfall twice or even two typhoons consecutively making landfall, and the topography is mostly mountainous and hilly, resulting in notable interactions between engineering and hydrological environments^18,19^, Especially in the granite eluvial soil area^20,21^. Therefore, it is necessary to pay attention to the scouring and erosion phenomena occurring on road slopes caused by heavy rainfall to prevent soil erosion in the construction of expressways, high-speed railways and other traffic projects in Guangzhou and surrounding granite residual soil areas.

The implementation of surface vegetation cover is a simple, economical and feasible slope protection method to control soil erosion and reduce soil loss and has been widely adopted^22–25^. Slope protection via vegetation is mainly realized by hydrological effects^26–32^. Vegetation intercepts rainfall, weakens splash erosion and inhibits surface runoff^33,34^. Under different climatic conditions, such as alpine, temperate, semiarid, tropical and other climate conditions, the influence of vegetation on the hydrological effect of slopes is highly different^31,35–37^. In various regions, such as the loess region of China, northern Mexico and the Canary Islands of Spain, the relationship between vegetation and slope hydrological effects exhibits different laws^25,38–40^. Similarly, the hydrological effects of different vegetation types, such as bovine sinew, alfalfa, wood and ginger, are also notably different^14,15,41,42,43^. The above research results confirm that vegetation exerts its slope protection impact through hydrological effects, and the effect is strongly influenced by climate, region and vegetation type.

The hydrological effect of vegetation slope under rainfall is generally studied by means of field monitoring or model (field, laboratory) tests. El Kateb et al.^44^ carried out the field experiments in the Shangnan County of China using 33 small erosion plots of 7 m^2^ in size to determine and compare the soil loss and surface runoff from five vegetation covers and three levels of slope gradient. The result reveals that farmlands generated the highest runoff and soil loss, whereas the tea plantations at slopes > 30° were most susceptible to erosion. Nine standard runoff plots located in the tableland-gully region of the Loess Plateau in China were chose to monitor surface runoff and soil loss. The experiments find that rainfall events with high intensity and short duration can cause more surface runoff and soil loss under all vegetation types, vegetation with high ground cover can Significantly reduce soil erosion^45^. The similar experiment had also found that the grass communities are more effective than shrub communities to enhance soil erosion resistance^46^. In the aspect of field model test, through the field simulated rainfall experiments, Zhang et al.^47^ points out that soil and water conservation function of grassland is direct sediment interception based on surface vegetation canopy for runoff and sediment regulation, the effect of shoots and roots on soil loss was almost equivalent to each other. Compare with grassland, grass & shrub land has the similar efficiency in bank slope erosion reduction, but is more suitable to control erosion in cutting slope^48^. The vegetation community succession also can significantly reduce the runoff velocity and power, and the development of plant root system significantly reduces the runoff volume^49^. In the aspect of laboratory model test, Zhao et al.^50^ made 8 slope models of 2 m × 0.5 m × 0.45 m with angle of 30° to determine the effect of rainfall intensity and vegetation cover on runoff volume, sediment load, and runoff hydraulics characteristics. The result shows that the runoff volume and sediment load of the bare plot are greater than those of vegetation-covered plots under three different rainfall intensities, the plot displayed the best performance for soil loss control when the *Cynodon dactylon* and *Indigofera amblyantha* applied together. The slope angle also has an important effect on erosion, which will have faster speed and heavier degree with the steeper slope angle^20^. In addition, Han et al.^51^ also considered the impact of vegetation coverage and rainfall intensity on slope runoff and sediment yield.

Compare with field monitoring and field test, laboratory model test method has advantages in time and operation, which will be used in the research, but it is critical to simulate the field conditions as much as possible, especially in artificial rainfall, slope soil filling and vegetation maintenance, etc. Through pre-investigation of the granite residual soil slope and common vegetation in northern Guangzhou, against the background of a certain typhoon making landfall twice or even two typhoons consecutively making landfall, this article selects *Zoysia japonica*, commonly applied as surface vegetation cover, and *Nephrolepis cordifolia*, a natural dominant vegetation, to perform a comparative study of the hydrological effects on covered and exposed slopes by means of indoor two-stage continuous high-rainfall model tests. It hopes to provide a basis for ecological slope protection in local traffic engineering construction under extreme rainfall conditions.

## Test design and measurement

### Model of the slope

**Figure 1:**
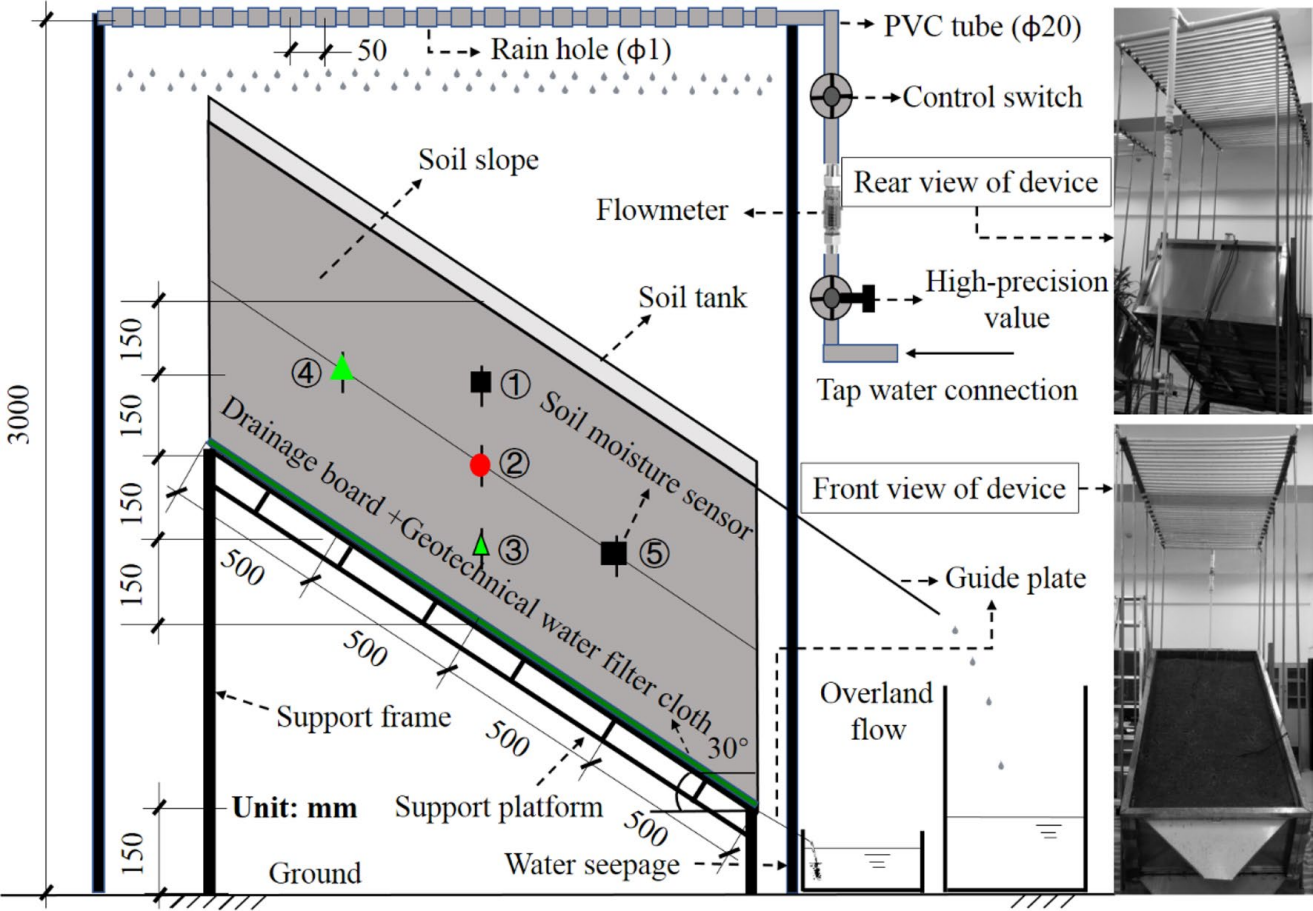
Test apparatus.

Three model soil boxes were constructed of stainless steel and tempered glass with a length of 200 cm, a width of 100 cm and a depth of approximately 65 cm. These soil boxes passed the tightness test. A support platform was located under the soil box to adjust the angle between the bottom of the soil box and the ground, and the angle was set to 30° in these tests, as shown in Fig. 1. The soil box was filled with granite residual soil collected from a northern suburb of Guangzhou, Guangdong Province. The soil is characterized by a high differentiation degree and a relatively high porosity ratio, and the basic physical property indexes are listed in Table 1. Before filling, a plastic drainage board was arranged at the bottom of the soil box, and a geotechnical water filter cloth was emplaced on top. Considering that granite residual soil naturally accumulates without external influences. The soil was first passed through a 5-mm sieve and then uniformly filled in four layers via natural accumulation with the multipoint method, which is a fixed-point and quantitative method, involving scraping, plate compaction and other operations. The thickness of each layer was 150 mm, and the total thickness of the slope was 600 mm. The density of the soil filling material was selected based on the natural density of the sample soil after natural air drying. The natural density of the sample soil was 1.65 g/cm^3^, and the natural water content reached 19.6%. The measured water content of the air-dried sample soil before filling was 7.7%, and the control density of the soil filling material was approximately 1.49 g/cm^3^, while the compaction degree approximately reached 91.2%. Soil moisture sensors were embedded when the soil fill reached the preset position. One of the three slope models was an exposed slope, and the other two slopes were vegetated slopes, which were planted with *Nephrolepis cordifolia* and *Zoysia japonica*. *Nephrolepis cordifolia* exhibits well-developed root systems and nodules. To avoid any slope soil effects during transplantation, the experimental soil was cultivated in advance. *Zoysia japonica* roots are short and shallow, and turf can be directly laid without advance cultivation. After completion of slope vegetation transplantation, watering and curing were performed over ≥ 60 days. To ensure the same initial soil moisture content in the three models, the exposed slopes were sprayed with the same amount of water. Before the tests, the soil moisture content in the three slopes at different depths was measured to range from 3.8 to 4.2%, which was relatively uniform.

**Table 1:** Physical property indexes of the test soil.

Specific gravity	Liquid limit (%)	Plastic limit (%)	Plasticity index	Maximum dry density (g/cm^3^)	Optimal moisture content (%)	Engineering classification
2.57	43.6	26.8	16.8	1.513	23.7	Sandy clay

### Artificial rainfall

In the experiments, three custom network-managed artificial rainfall devices were adopted to simulate rainfall, which were independent and capable of simultaneous operation. Each rainfall device was constructed of PVC water pipes, 3 m in height and 20 mm in diameter, which were connected with tee-shaped and right-angle elbow connectors. The rain hole diameter was 1 mm, and the spacing was set to 50 mm. The rainfall intensity achieved by the artificial rainfall device was controlled by a flowmeter and a precision-controlled copper valve, and the control range was 30–300 mm/h, which met the rainfall parameter setting requirements. Before each test, the rainfall characteristics of the device were tested as follows: raindrops were collected in a 1000-mL graduated cylinder within 1 min at four randomly selected positions below the rainfall device, and the number of raindrops was recorded. During the test, it was assumed that the rainfall intensity was the same and that the raindrops were spherical. The test results listed in Table 2 indicate that the custom device attained a good rainfall uniformity and stability, which satisfied the test requirements^22,41^.

**Table 2:** Test data of the artificial rainfall simulator.

Test location	1	2	3	4
Total mass of rainfall (g)	20.36	21.06	20.79	21.48
Number of raindrops (drops)	99	100	101	101
Raindrop weight (g)	0.2057	0.2106	0.2058	0.2127
Raindrop radius (cm)	0.3662	0.3691	0.3663	0.3703

In the experiments, the heavy rainfall caused by the four typhoons making landfall in South China from 2015 to 2018, including Typhoons Rainbow (2015), Nida (2016), Hato (2017) and Mangosteen (2018), was simulated. The effective rainfall intensity of a typhoon was calculated as follows: when Typhoon Rainbow made landfall, the rainfall was approximately 500 mm, and assuming that the effective rainfall duration during this typhoon event was 4 h, the effective rainfall intensity was calculated as 125 mm/h. Similarly, the 24-h rainfall due to Typhoon Nida ranged from approximately 200–350 mm, and the effective rainfall intensity ranged from 50 to 87.5 mm/h. The 24-h rainfall due to Typhoon Hato reached 100–200 mm, and the effective rainfall intensity was 25– 50 mm/h. The 24-h rainfall due to Typhoon Mangosteen was 250–400 mm, and the effective rainfall intensity ranged from 50 to 80 mm/h. According to the effective rainfall intensity of the above typhoons, two rainfall events under the same rainfall amount and different rainfall intensities were simulated in the experiments considering that in 2018, Typhoons Bailiga and Mangkhut successively made landfall in South China, with an interval of approximately 80 h. In 2019, Typhoon Wipa made landfall for the second time, with an interval of nearly 18 h. In this paper, 48 h was chosen as the rainfall interval, which is an approximate average value. The specific parameters are summarized in Table 3.

**Table 3:** Characteristics of the artificial rainfall.

Rainfall process	First stage	Second stage
Interval time	48 h	
Rainfall intensity (mm/h)	75	150
Rainfall duration (min)	60	30
Rainfall amount (mL)	100,000	

### Measurement quantities

The test measurements included the following three items: (1) Internal volumetric slope water content. MS-10 soil moisture sensors produced by Dalian Zheqin Technology Co., Ltd., were adopted to measure the average volumetric soil water content involving a cylinder with a diameter of 7 cm and a height of 7 cm centered on the central probe of each sensor. The sensors were arranged along two directions, including the vertical direction in the middle of the slope, with 3 sensors arranged at an interval of 150 mm, as denoted by ①, ②, and ③ in Figs. 1, and 3 sensors arranged at an interval of 500 mm in the middle of the soil layer along the slope centerline, numbered as ④, ② and ⑤. During the rainfall test, the data collection frequency of the moisture sensors was set to 1 min/time. After the rainfall test, the collection frequency was set to 10 min/time. (2) Surface runoff and sediment contents. Slots were located along the lower side of the box wall at the level of the slope, and a diversion plate was placed parallel to the slope surface. The initial surface runoff time was recorded, and a container was employed for runoff collection and weighing purposes. If the surface runoff contained any sediment, the sediment in the container was allowed to settle after the rainfall test. The sediment was weighed after dewatering and drying, and the water quality was then determined by subtracting the sediment mass from the total mass. (3) Rain seepage amount. As rainwater seeped toward the bottom of the slope body, it was discharged from the bottom of the soil box through the geotextile filter cloth and drainage plate. The bottom of the lower side box wall was slotted, and a diversion plate was placed parallel to the bottom. The start time of rainwater seepage was recorded, and a container was used to collect rainwater and determine the seepage amount via weighing. To ensure accuracy of the abovementioned measurement quantities, the simulated rainfall was prevented from falling directly into the collection container during the tests.

## Test results and analysis

### Comparative analysis of the surface runoff

Under the condition of the same rainfall intensity, the surface runoff characteristics of the three vegetation-covered slope models exhibit notable differences. The surface runoff occurrence time, total amount and sediment content of each slope model during the two rainfall processes are listed in Table 4.

**Table 4:** Surface runoff statistics.

Rainfall process	First stage (75 mm/h)			Second stage (150 mm/h)		
Vegetation type	Exposed	*Nephrolepis cordifolia*	*Zoysia japonica*	Exposed	*Nephrolepis cordifolia*	*Zoysia japonica*
Surface runoff occurrence time	No surface runoff	No surface runoff	42 min	0 min	No surface runoff	3 min
Amount of surface runoff	0	0	13,540.5 g	92,357 g	0	49,197.5 g
Sediment content	0	0	0	23,366.5 g	0	31 g
Rainwater content	0	0	13,540.5 g	68,990.5 g	0	49,166.5 g

**Figure 2:**
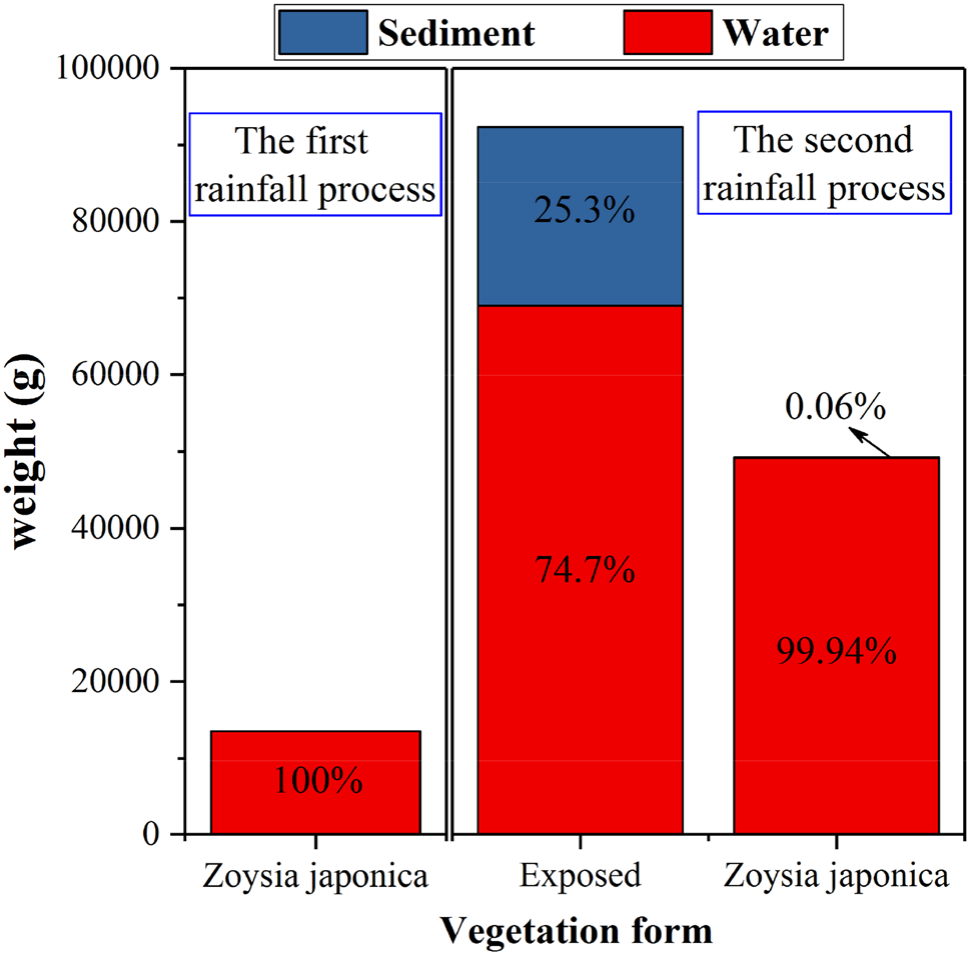
Surface runoff amount and proportion.

**Figure 3:**
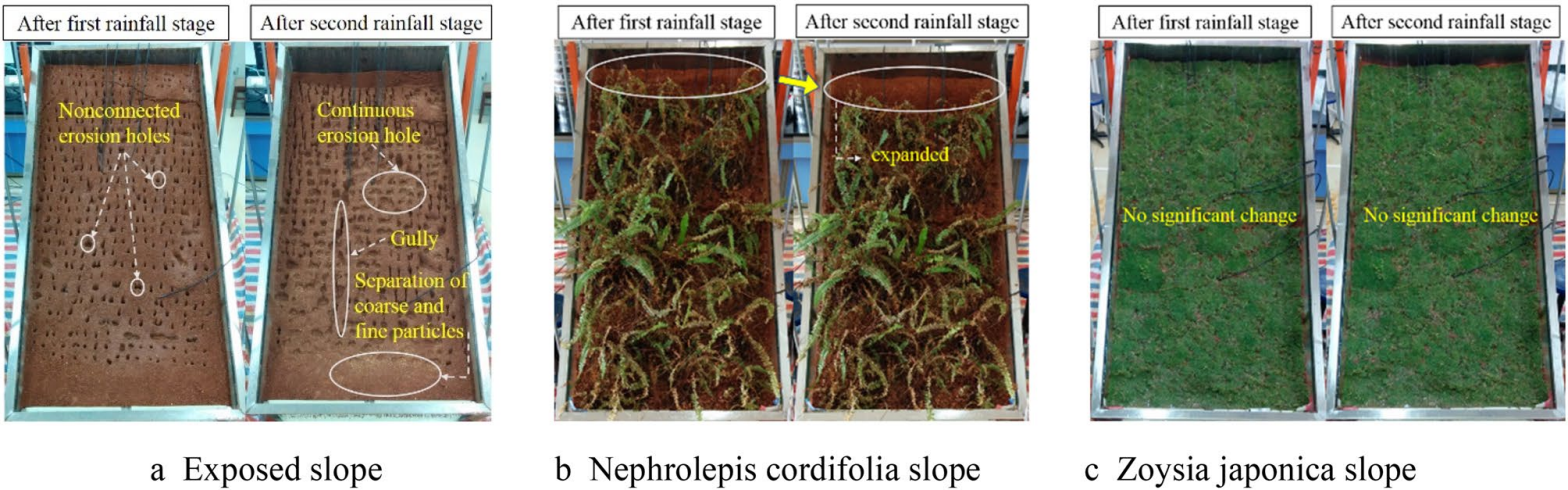
Comparison of the slope surfaces at the end of the rainfall test.

The above table indicates that the exposed slope did not experience surface runoff in the first rainfall stage, and complete rainfall infiltration occurred. However, many individual soil pores were observed on the slope surface under the combined action of rain splashing and infiltration. Surface runoff occurred at the beginning of the second rainfall event, which indicates that the increase in soil moisture content and the seepage compaction effect due to rainfall in the first stage greatly reduced the slope permeability. In the case of secondary rainfall and a higher intensity, complete rainwater infiltration did not occur from the beginning, resulting in surface runoff. In the entire rainfall process, the total surface runoff amount was almost equal to the total rainfall amount, accounting for 92.4% of the total rainfall amount, and the sediment content was high, reaching 25.3%, as shown in Fig. 2. Compared to the end of the first rainfall stage, the erosion holes observed on the slope surface were no longer disconnected at the end of the second rainfall stage but appeared as continuous patterns and even formed gullies across the entire slope from top to bottom, which caused more serious damage on the surface. Surface runoff also resulted in the separation and stratification of coarse and fine soil particles in the lower part of the slope body, as shown in Fig. 3a. The *Nephrolepis cordifolia* slope did not exhibit surface runoff, and complete rainwater infiltration was observed. However, compared to the end of the first rainfall stage, the exposed area at the top of the slope expanded after the second rainfall stage, indicating that the slope experienced a certain sliding displacement and demonstrated signs of instability, as shown in Fig. 3b.

Surface runoff occurred in the two rainfall processes on the *Zoysia japonica* slope. However, surface runoff occurred at 42 min after rainfall initiation in the first stage, which was much later than that observed in the second stage, just 3 min after the start. *Zoysia japonica* contains very dense branches and leaves near the ground, which is beneficial for rainwater confluence deceleration. This also indicates that rainwater does not converge along the slope until the water layer formed between the stem leaf reaches a certain height. In the initial rainfall stage, due to the low water content, the rainwater infiltration rate was much higher than that under the condition of a low rainfall intensity, and complete rainwater infiltration occurred. As the soil water content gradually increased, both the storage capacity of the slope soil and the rainwater infiltration rate decreased. When the rainwater infiltration rate was lower than the rainfall rate, the height of the water layer between the stem leaf increased, and finally, confluence was observed. However, this whole process occurred relatively slowly. At 42 min, the surface runoff amount was 13,540.5 g, accounting for 75.2% of the total rainfall amount during the corresponding period, which indicates that once confluence is established, surface runoff becomes the main hydrological effect without sediment. After the end of the first rainfall stage, the dense aboveground stem leaf and underground root system of *Zoysia japonica*, as well as the low slope permeability at this time, suitably maintained the water layer between the stem leaf. As a result, the confluence time was greatly shortened after the beginning of the second higher-intensity rainfall stage. This was reflected by the quick occurrence of surface runoff after rainfall initiation. The total runoff amount accounted for approximately 50.7% of the total rainfall amount, of which the sediment content was only 0.06%, as shown in Fig. 2. Although the characteristics of surface runoff caused by the two rainfall events differed, due to the multiple protective effects of *Zoysia japonica* stem and leaf blocking, and root reinforcement^1,2^, the slope surface did not notably change in the entire rainfall process, as shown in Fig. 3c.

### Variation analysis of the volumetric soil moisture content

After the rainfall process begins, some rainwater infiltrates the soil along the direction of the soil depth. The volumetric soil moisture content at different depths successively increases. As the rainfall process continues, the soil water content gradually established a balance between infiltration and seepage. Moreover, the maximum volumetric moisture content is reached. The equilibrium state mainly depends on the void content in the soil. The higher the void ratio, the more difficult it is to reach the equilibrium state, and the shallower the soil layer buried depth, the shorter the time required to reach the equilibrium state. After the end of rainfall, water seepage in the soil layer continues, but due to the gradual decrease in infiltration to zero, the volumetric soil moisture content continues to decrease and eventually tends to remain stable. In addition, the soil is unsaturated, and a new equilibrium state is attained. The equilibrium state not only depends on the void characteristics of the soil but also depends on the atmospheric temperature and humidity, and the equilibrium state constantly changes according to these aspects^52^.

**Figure 4:**
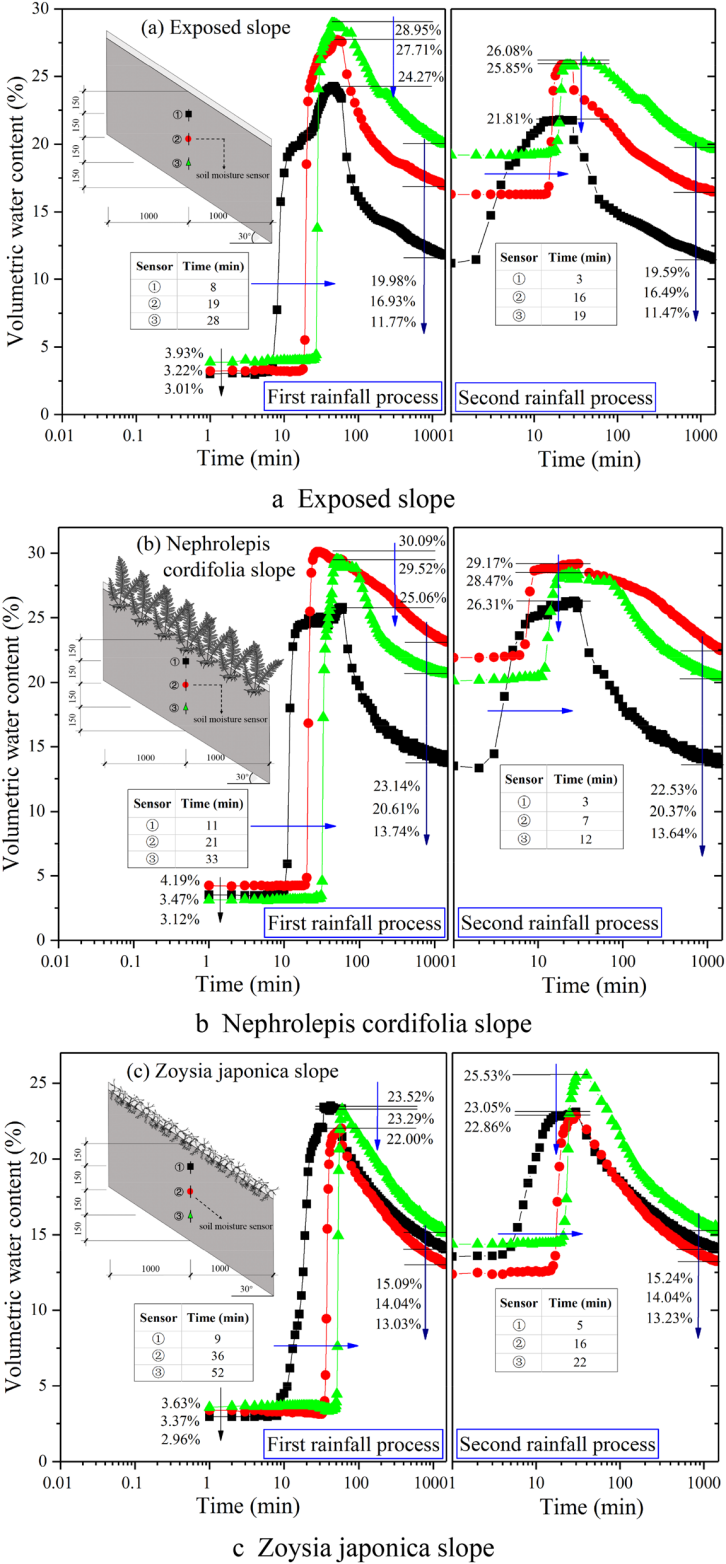
Variation law of the soil moisture content at sensor locations ①–③ on each slope.

Figure 4 shows the test data of sensors ①, ②, and ③ on the three slopes, namely, the change curve of the volumetric soil moisture content at the same location but different depths with the rainfall process in semilogarithmic coordinates. The values of the three sensors remained basically unchanged in the initial stage of the first rainfall process and then rapidly increased from shallow to deep. However, the corresponding start time of the aforementioned sudden change phenomenon greatly varied among the different vegetation types. Specifically, on the exposed slope, the sudden change occurred at 8, 19 and 28 min, respectively, while on the *Nephrolepis cordifolia* slope, it was observed at 11, 21 and 33 min, respectively, and on the *Zoysia japonica* slope, it occurred at 9, 36 and 52 min, respectively. Obviously, vegetation decreased the rainwater infiltration rate due to the interception effect of the stem leaf and the blocking effect of the roots. Compared to the exposed slope, the sudden change on the *Nephrolepis cordifolia* slope lagged 3, 2 and 5 min, respectively, from shallow to deep depths, and the lag degree basically remained the same. In contrast, at the corresponding positions on the *Zoysia japonica* slope, the lag times were 1, 17 and 24 min, respectively, and the lag degrees at depths of 300 and 450 mm were much higher than that at a depth of 150 mm. This indicates that the action mechanism of these two kinds of vegetation in terms of rainwater infiltration inhibition is obviously different. The inhibitory effect of *Nephrolepis cordifolia *is more comprehensive, indicating that the control action mainly originates from above the ground. In contrast, the inhibitory effect of *Zoysia japonica* is local, reflected by the obvious inhibitory effect at a depth of 300 mm or shallower, which is very similar to that observed on the exposed slope above this depth. This demonstrates that the inhibitory effect mainly originates from a certain depth range below the surface. When rainwater infiltrates to a certain depth, the soil moisture content at this depth rapidly increases and reaches a peak at the end of the first rainfall process. On the exposed and *Nephrolepis cordifolia* slopes, the peak water content is the lowest at the location of sensor ① and higher at sensors ② and ③, but the difference is not obvious among these sensor locations. At the corresponding positions on the *Nephrolepis cordifolia* slope, the moisture content is slightly higher than that in the exposed slope, by approximately 1–1.5%. This indicates that *Nephrolepis cordifolia* slightly enhances the soil water storage capacity, but the change in the void structure of the soil remains very limited overall. On the *Zoysia japonica* slope, the water content peak values at the three depths are relatively similar, and the values at sensors ② and ③ are notably lower than those in the other two slopes. This further verifies the notable inhibitory effect of *Zoysia japonica* on rainwater infiltration. However, the rainfall amount in the first stage is not enough to fully saturate the soil at sensors ② and ③. Upon the end of the first rainfall stage, rainwater seepage continues, and the moisture content at each depth decreases in a similar manner, initially fast and then slowly. After 48 h, the water content in the exposed slope is the highest, namely, 19.98% at the location of sensor ③, and the lowest value of 11.77% is observed at sensor ①. On the *Nephrolepis cordifolia* slope, the highest water content reaches 23.14% at the location of sensor ②, and at sensor ①, the lowest value is found, at 13.74%. The water content in the Zoysia slope at the location of sensor ③ is 15.09%, which is slightly higher than that at the locations of sensors ① and ②, at 14.04% and 13.03%, respectively. The moisture content distribution is always relatively uniform. In comparison, the root system of *Nephrolepis cordifolia* generates more obvious inhibitory effects on water infiltration and soil water loss.

**Figure 5:**
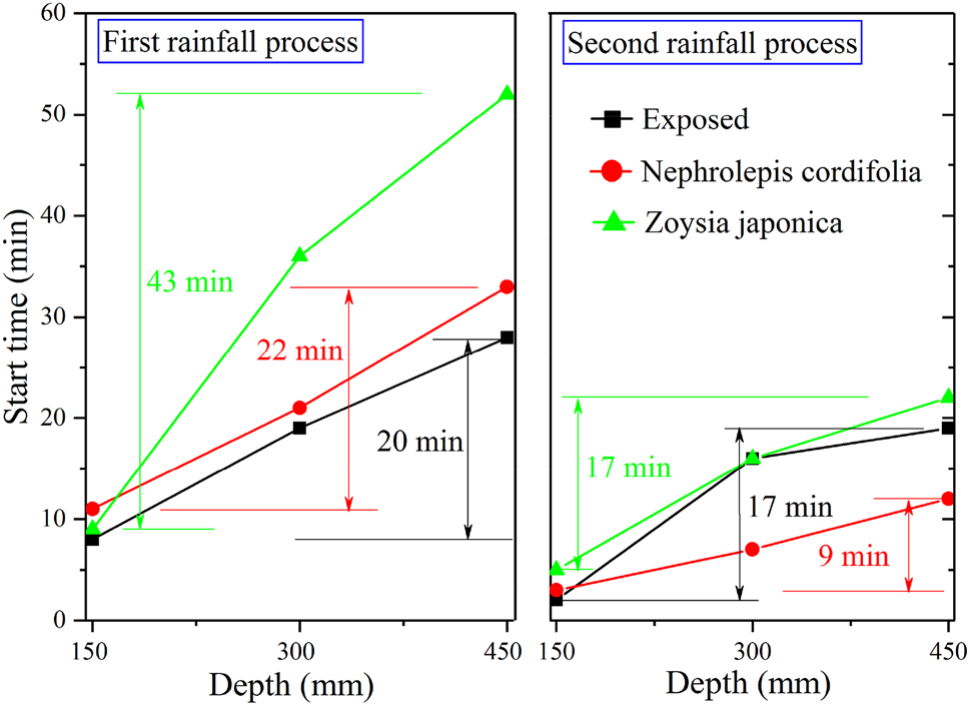
Comparison of the initial time difference of rainwater infiltration at the various depths.

Overall, the pattern observed at the three sensor locations in the second rainfall stage is similar to that observed in the first stage. The main difference pertains to the corresponding time when the water content significantly increases and the achieved peak water content. The specific performance is as follows: after the first rainfall stage, the soil moisture content increases, and the established rainwater seepage channels are more continuous. As a result, the time required for rainwater seepage to reach the same depth in the second stage is greatly reduced. As shown in Fig. 5, on the three slopes, approximately 10 min are required for rainwater seepage to reach the depth of sensor ① in the first stage but no more than 5 min are required in the second stage. The required time is greatly shortened for rainwater to seep from the depth of sensor ① (depth = 150 mm) to that of sensor ③ (depth = 450 mm). For example, the time on the *Nephrolepis cordifolia* slope is reduced from 22 to 9 min and that on the *Zoysia japonica* slope is reduced from 43 to 17 min. Although the rainfall intensity is increased in the second stage, due to the large amount of surface runoff generated on the exposed slope, the actual rainfall infiltration is much lower than that in the first stage. This results in a decrease in the peak water content measured by each sensor in the second rainfall stage, which is approximately 2–3%. This phenomenon also occurs on the *Nephrolepis cordifolia* slope, with a difference of approximately 1%, but the reason is not a smaller infiltration amount but rather a larger discharge amount. In contrast, the performance of the *Zoysia japonica* slope is different. The peak value at sensor ① slightly decreases by approximately 0.5%, and that at sensors ② and ③ increases by approximately 1% and 2%, respectively. This occurs because the soil at the depths of sensors ② and ③ has not yet reached complete saturation in the first stage.

**Figure 6:**
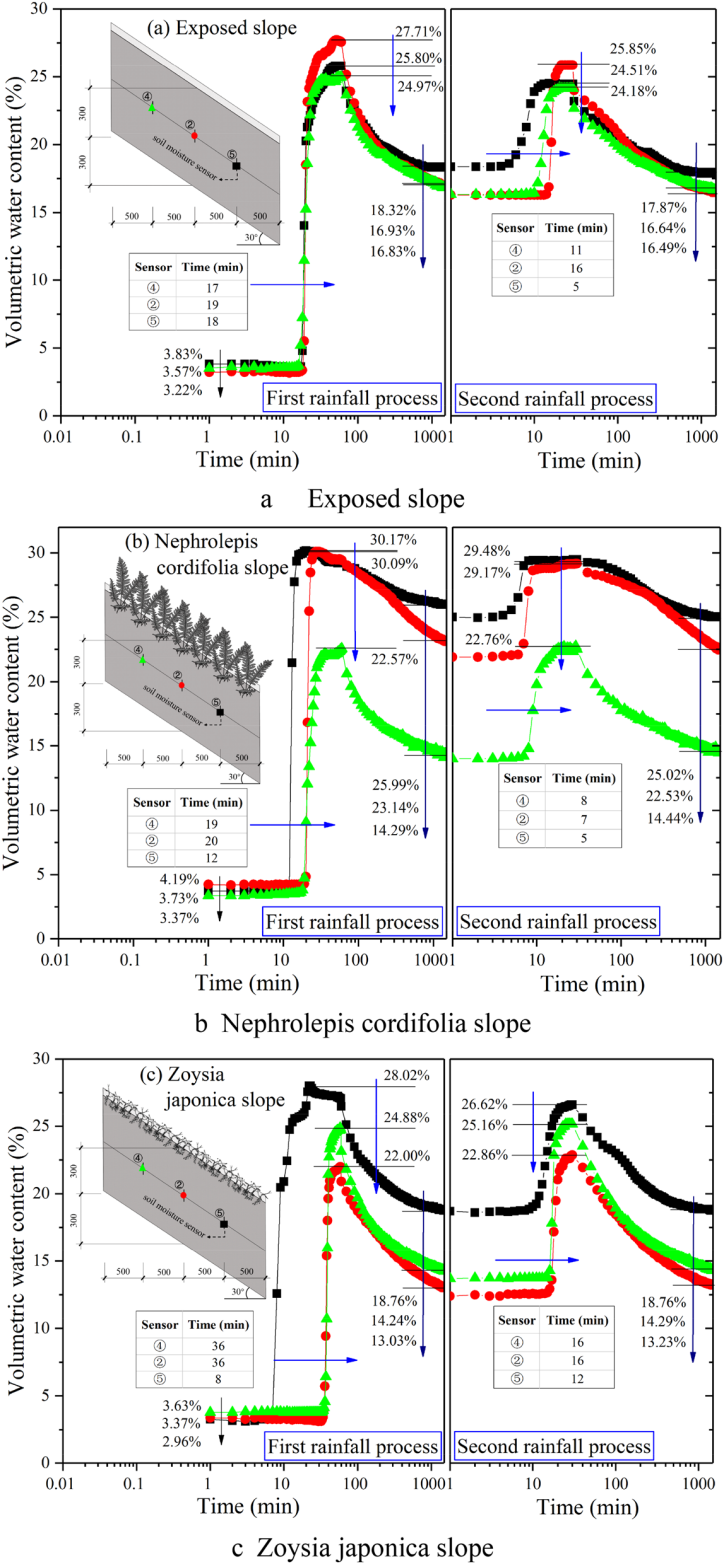
Variation in the soil moisture content at the depths of sensors ②, ④ and ⑤.

Figure 6 shows the test data of sensors ④, ②, and ⑤ on the three slopes, namely, the change curve of the volumetric soil moisture content in semilogarithmic coordinates at the same depth but different locations during the rainfall process. The above figure shows that the variation rules at the three sensor locations on the exposed slope basically coincide in the first rainfall stage. This also indirectly verifies that the filling and rainfall simulation procedures in this model test exhibit a good uniformity. However, at the end of the first rainfall stage, the moisture content in the lower-slope area is approximately 1.5% higher than that in the middle- and upper-slope areas. In the second rainfall stage, the soil water content at the three locations notably increases, and the corresponding initial time is also different, which is the shortest in the lower part of the slope (5 min) and the longest in the middle part of the slope (16 min), as shown in Fig. 6a. In the first stage, the initial time corresponding to the obvious increase in soil water content is 12 min in the lower part of the *Nephrolepis cordifolia* slope, which is much earlier than that observed in the middle (20 min) and upper (19 min) parts of the slope. The corresponding time in the second stage is shortened by more than half to 5, 7 and 8 min. After the end of rainfall, the moisture content at the lower- and mid-slope positions exhibits a slow decline, which is different from that at the upper-slope position. After 48 h, the soil water content in the lower-slope part is nearly 26%, which is approximately 7.5% higher than that in the exposed slope, as shown in Fig. 6b. The initial time corresponding to the obvious increase in soil moisture content on the *Zoysia japonica* slope is also the shortest at the lower-slope position (8 min) in the first stage, and at both the mid- and upper-slope positions, the time is 36 min. In the second stage, the corresponding time at the lower-slope position is extended to 12 min, and that at the mid- and upper-slope positions is reduced to 16 min. In the two rainfall processes, the peak water content is the highest in the lower part of the slope, followed by that in the upper part of the slope, whereas the peak water content in the middle part of the slope is the lowest. The peak water contents at all corresponding positions decrease in the second stage, and the decrease observed at the lower-slope position is the most obvious, at approximately 1.5%. After rainfall, the decrease rate of the moisture content is slightly slower in the lower part of the slope, and finally, it is maintained at a level equivalent to that on the exposed soil slope after 48 h. However, the water content in the middle and upper parts of the slope is much lower than that on the exposed slope, approximately from 2.5 to 3.5%, as shown in Fig. 6c. Generally, after rainfall initiation, the soil water content at the same depth first increases in the lower part of the slope. After rainfall, the water content corresponding to the same time is also the highest in the lower part of the slope, which is in accordance with the general law. The reason is that the soil in the lower part of the slope not only experiences rainwater infiltration along the vertical direction but also experiences rainwater infiltration along the upward direction.

### Comparative analysis of the slope permeability and water storage capacity

The initial time and total amount of rainwater seepage on the three slopes in the two rainfall stages are listed in Table 5.

**Table 5:** Rainwater seepage comparison.

Vegetation type	Exposed	*Nephrolepis cordifolia*	*Zoysia japonica*	Exposed	*Nephrolepis cordifolia*	*Zoysia japonica*
Rainfall process	First stage (75 mm/h)	Second stage (150 mm/h)				
Seepage initiation time (min)	43	22	25	10	3	4
Seepage amount (mL)	91,150	60,240	77,300	22,400	97,600	39,250

**Figure 7:**
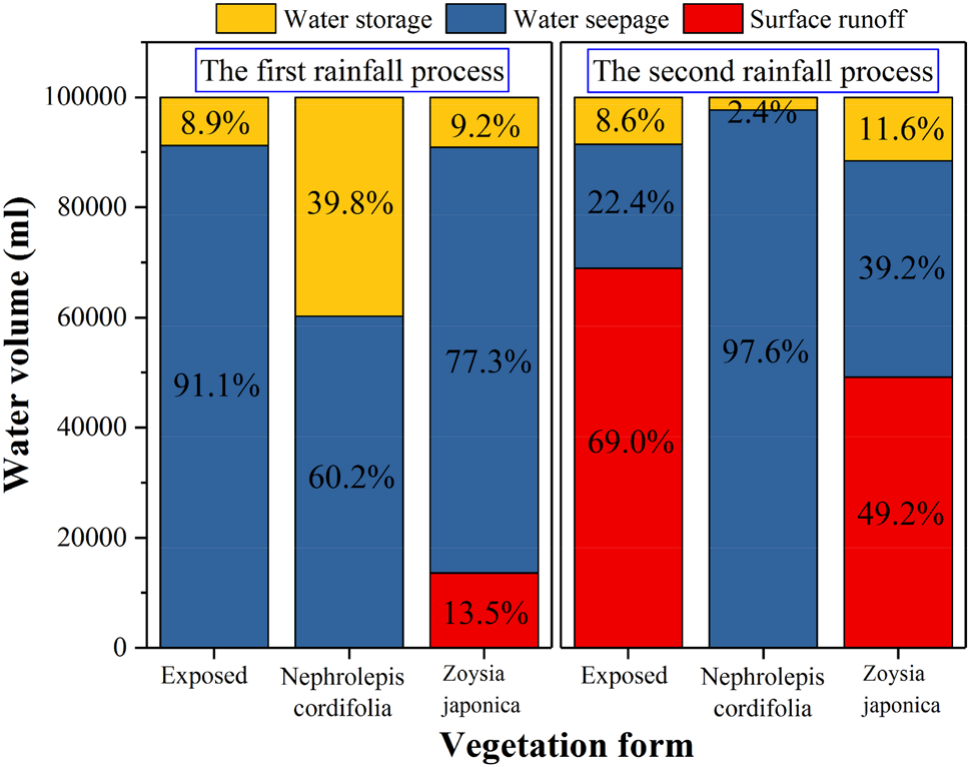
Distribution ratio of the rainfall patterns of the different vegetation-covered slopes.

Rainwater seepage in the first rainfall stage generally occurs later than that in the second stage. This is related to the initial water content of the slope soil before rainfall, and the initial soil water content in the second rainfall stage is much higher than that in the first rainfall stage. Therefore, the time required to reach the discharge condition is notably shorter than that required in the first stage. The rainwater seepage times on the two vegetation-covered slopes are roughly the same in the two rainfall processes, and both are shorter than that on the exposed slope. This indicates that the presence of vegetation improves the slope permeability. In terms of the rainwater infiltration amount, in the first rainfall process on the exposed and *Zoysia japonica* slopes, the amount is much larger than that in the second rainfall process, while the opposite is observed on the *Nephrolepis cordifolia* slope. To evaluate the permeability characteristics of the three model slopes intuitively and quantitatively, the equivalent permeability coefficient *k* is calculated by *Vl*/(*A*∆*ht*),where *V* is the rainfall discharge volume, mm^3^; *t* is the time difference between the start of rainwater discharge to the end of rainfall, s; *l* is the length of the seepage path, with the vertical soil thickness set to 591.6 mm; ∆*h* is the water head difference, which is equal to the length of the seepage path, mm; and *A* is the cross-sectional area of the horizontal plane of the slope, which is *A* = 8.1 × 10^5^ mm^2^. The calculation results are listed in Table 6. In the first rainfall stage, the equivalent permeability coefficient of the exposed slope is slightly higher than that of the *Zoysia japonica* slope. The equivalent permeability coefficient of the *Nephrolepis cordifolia* slope is the lowest, at approximately 64% of that of the exposed slope. Combined with the proportion of surface runoff, discharge volume and water storage capacity of the different vegetation-covered slopes, as shown in Fig. 7, there is no surface runoff on the exposed slope in the first stage, i.e., complete rainwater infiltration occurs, and approximately 91.1% of the rainwater is finally discharged within the shortest time. Therefore, the equivalent permeability coefficient is the highest. The surface runoff observed on the *Zoysia japonica* slope accounts for approximately 13.5% of the total rainfall amount, and rainwater infiltration accounts for 77.3% of the total rainfall amount, which is lower than that on the exposed slope. Moreover, the seepage time on the *Zoysia japonica* slope is slightly longer, and the equivalent permeability coefficient is slightly lower than that of the exposed slope. The discharge time on the *Nephrolepis cordifolia* slope is almost the same as that on the *Zoysia japonica* slope. Although the former exhibits no surface runoff, rainwater seepage accounts for 60.2% of the total rainfall amount, which is lower than that observed on the latter, at 77.3%. Therefore, the equivalent permeability coefficient of the *Nephrolepis cordifolia* slope is the lowest. In the second stage, surface runoff on the exposed slope accounts for approximately 69% of the total rainfall amount. As a result, the seepage volume is greatly reduced, and the equivalent permeability coefficient is also reduced to only 25% of that in the first rainfall stage. For the same reason, on the *Zoysia japonica* slope, discharge accounts for 39.2% of the total rainfall amount, which is much lower than that in the first stage, at 77.3%. The equivalent permeability coefficient decreases to approximately 50% of that in the first stage. The *Nephrolepis cordifolia* slope still exhibits no surface runoff in the second stage, but the seepage volume increases to 97.6% of the total rainfall amount. Therefore, the equivalent permeability coefficient does not decrease but rather increases, namely, to 1.67 times that in the first stage.

**Table 6:** Equivalent permeability coefficient.

Rainfall stage	Exposed slope (m/s)	*Nephrolepis cordifolia* slope (m s^-1^)	*Zoysia japonica* slope (m/s)
First stage	1.3 × 10^−6^	$8.4\times \hspace{0.17em}$10^−7^	$1.1\hspace{0.17em}\times \hspace{0.17em}$10^−6^
Second stage	3.2 × 10^−7^	$1.4\times \hspace{0.17em}$10^−6^	$5.5\hspace{0.17em}\times \hspace{0.17em}$10^−7^

**Figure 8:**
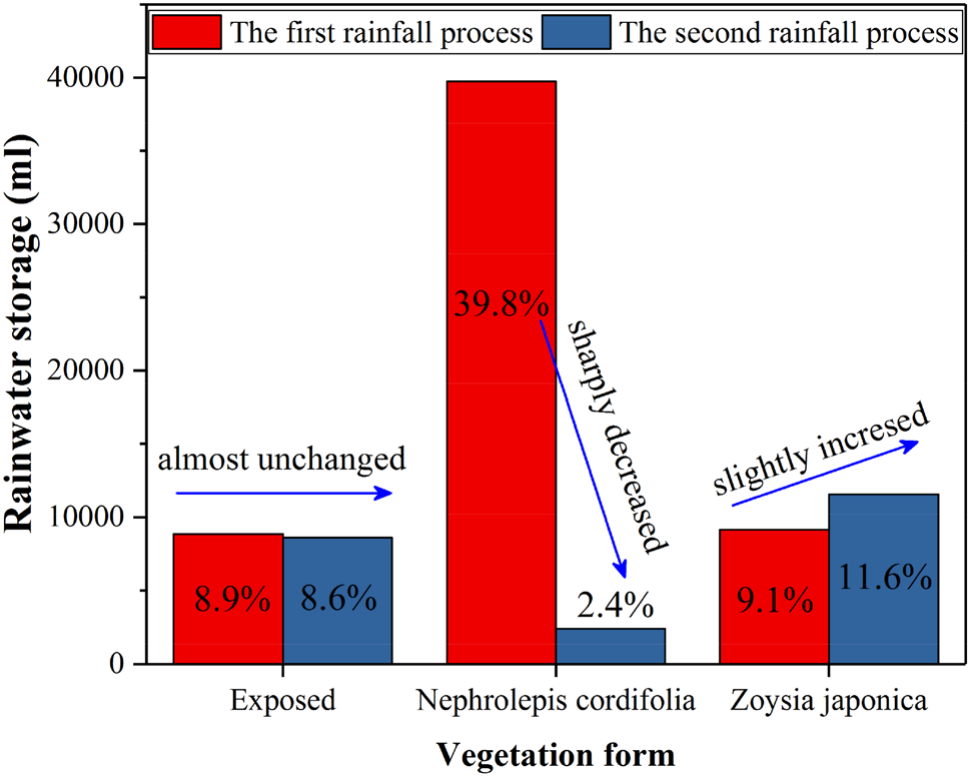
Comparison of the water storage capacity of the different vegetation-covered slopes.

Figure 8 shows that in the first rainfall stage, the water storage ratio of the exposed and *Zoysia japonica* slopes is roughly the same, at approximately 9% of the total rainfall amount. The water storage ratio of the *Nephrolepis cordifolia* slope reaches nearly 40%, which indicates a much higher water storage capacity than that of the former two slopes. In the second stage, the water storage ratio of the exposed slope remains unchanged, while that of the Zoysia slope only slightly increases, from 9.1 to 11.6%. This demonstrates that early rainfall exerts very little effect on the ability of these two slopes to absorb the subsequent rainfall. Rainwater on these slopes seeps and drains within a short period (within 48 h) after the end of the first rainfall stage, and the water storage capacity is restored. This also reflects the poor water storage capacity of these two slopes. In contrast, the *Nephrolepis cordifolia* slope only stores 2.4% of the rainfall amount in the second stage, which is much smaller than that stored in the first rainfall stage, at 39.8%. The *Nephrolepis cordifolia* slope does not discharge much water during the interval between the two rainfall events, and its water storage capacity is very high. This results in a very small water storage increment of this slope in the second stage.

## Discussion

**Figure 9:**
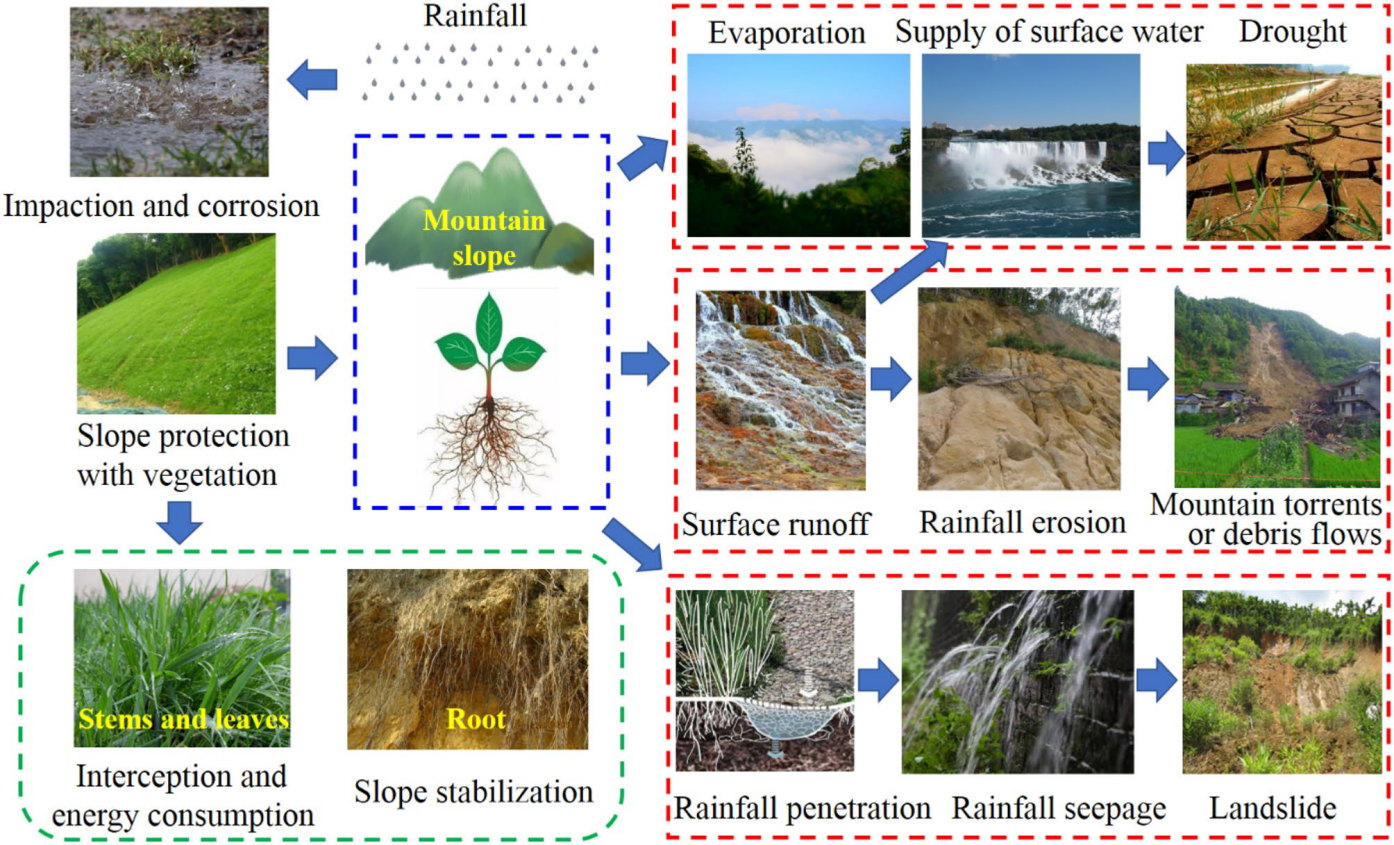
Rainwater shape on the vegetation-covered slopes due to rainfall and its results.

The whole rainfall process on a given slope is shown in Fig. 9. The first step comprises impaction and splash erosion on the slope. However, on slopes covered with herbaceous vegetation, the interception effect of the aboveground stem leaf greatly alters the speed and size of raindrops, consumes their energy, and greatly reduces the effects of impaction and splash erosion. Subsequently, rainwater appears in three forms. The first form is water vapor, produced via evaporation or transpiration from vegetation^53,54^. However, during the rainfall period, for the reasons of temperature and time, the proportion of this form is very small, and the impact on the slope is almost negligible. The second form is infiltrated rainwater, which leads to the gradual increase in slope soil water content from shallow to deep, in addition to the soil weight, and this generally weakens the mechanical properties. All these aspects are detrimental to the slope stability^55^. There are two infiltration directions, namely, the vertical and downhill directions. The former yields a compaction effect on the soil and changes the soil pore characteristics to a certain extent. The latter increases the sliding force of the slope, which does not facilitate a stable slope. When seepage reaches the lower boundary of the slope, some rainwater is discharged from the slope soil to form surface water. If rainwater is not discharged or the discharge amount is smaller than the infiltration amount, rainwater gathers inside the slope soil, and the pore water pressure increases. This further weakens the mechanical properties of the slope soil, increases the sliding force of the slope, reduces the slope stability and even causes landslide disasters^56^. The third form is surface runoff. When the rainwater infiltration amount is smaller than the rainfall amount per unit time, rainwater gathers on the surface and flows down the slope to form surface runoff. Surface runoff imposes erosion effects on slopes, including soil erosion, transportation and deposition processes. Finally, gullies of different sizes are formed on the slope, which leads to extreme geological disasters such as mountain torrents and debris flows^57^.

On vegetation-covered slopes, the stem leaf and root system of vegetation highly affect the above three forms of rainwater^26,58,59^. However, because the first form can be ignored in terms of the slope stability during rainfall, the hydrological effect of vegetation-covered slopes is mainly examined based on the changes in the latter two forms of rainwater. The second form of rainwater infiltration mainly depends on the pore characteristics of soil. Existing studies have shown that vegetation roots can significantly change the pore structure characteristics of soil^60–62^. Such change can be largely reflected by the variation of soil–water characteristic curve (SWRC), especially under wetting conditions. Leung et al.^63^ took shrub (Schefflera hepataphylla) as the object and proved that root induction dominates the variation of SWRC. However, this change is affected by many factors, and Ng and his team have carried out a lot of pioneering work on this, including considering soil density^64^, vegetation planting density^65^, root growth and decay^66^, and so on. Jotisankasa & Sirirattanachat^67^ studied the root system of vanilla roots and showed that the soil type (clayey sand, low plastic silt) was also an important factor. Therefore, it is extremely complex of the influence mechanism of roots on SWRC and even pore structure characteristics, especially the spatial distribution of roots of different vegetation in soils with different properties is highly uncertain and difficult to be described. Lu et al.^68^ also pointed out that the parameters related to the soil hydrological effect controlled by root distribution are highly uncertain, which is an important obstacle that makes it difficult to consider this effect in the problem of site scale. This paper analyzes the difference of rainwater infiltration reflected by different slopes only from the main characteristics of the roots of two plants. Any water arriving at the surface may infiltrate, but if the infiltration capacity of the soil is not satisfied, runoff will be generated at the surface. Surface vegetation has an important inhibiting effect on the third form, which is carried out in stages. The first stage is mainly reflected in the interception and buffering of plant stem leaf, and its influencing factors include vegetation type, stem and leaf biomass, etc.^69–71^. Once the storage components of are filled, however, water will either start to drip off the leaves and branches and reach the ground, or cascade on to lower leaves or branches. Or it will flow along the branches and down the plant stems as stemflow, reaching the ground in some concentrated form around the base of the vegetation and beginning to enter into the second stage^72^. The effect of this stage is realized through the main stem of the plant, ground root system and litters^73^. The surface roughness can comprehensively reflect these factors, and the higher the roughness, the stronger the inhibition effect on surface runoff^74,75^.

**Figure 10:**
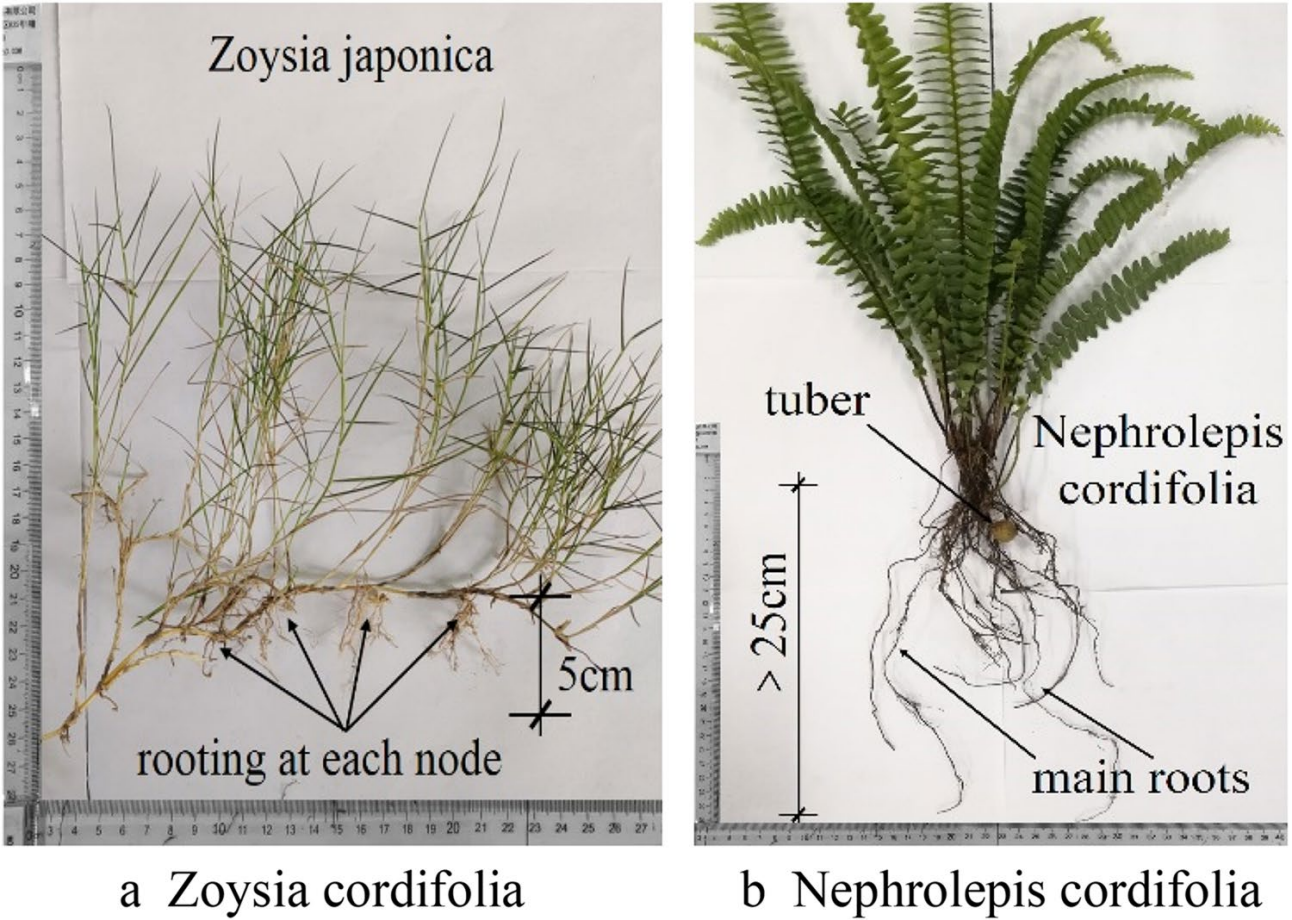
Roots of two plants.

In this experiment, compared to the exposed slope, the *Zoysia japonica* slope had a significant regulating effect on the second and third types of rainwater forms. As shown in Fig. 7, in the first rainfall stage, no surface runoff was observed on the exposed slope, and the surface runoff observed on the *Zoysia japonica* slope accounted for 13.5% of the total rainfall amount. In the second rainfall stage, the former accounted for 69% of the surface runoff amount, causing serious surface erosion damage, while the latter accounted for 49.2% of the surface runoff amount, with no obvious slope effect. *Zoysia japonica* exhibits slender stalks, tufted leaves, which are lanceolate, thick, hard and nearly leathery. The height of the stem leaf of *Zoysia japonica* is smaller than *Nephrolepis cordifolia*, approximately 5–10 cm. *Zoysia japonica* does not intercept rainwater multiple times, and the ability to absorb raindrop energy is also limited. As a result, rainwater reaches the surface and infiltrates soon after rainfall initiation. Below the ground, *Zoysia japonica* stem possesses short and dense internodes, with each node exhibiting the characteristic of rooting, so the root system is very well developed, but only within 5 cm below the surface, as shown in Fig. 10a^76^. This also indicates that the change in soil pore structure due to the *Zoysia japonica* root system is highly notable near the surface, which can greatly reduce the infiltration of rainwater and promote more rainwater to collect on the surface to form surface runoff. Therefore, the blocking effect of the root system plays a leading role in the inhibitory mechanism of *Zoysia japonica* on rainwater infiltration, which leads to a decrease in the rainwater infiltration rate and causes more rainwater to gather on the surface to form surface runoff. In the two rainfall stages of the experiment, surface runoff on the *Zoysia japonica* slope supports this finding. However, the thick, hard and tufted stem leaf of *Zoysia japonica* notably blocks surface runoff and reduces the flow velocity and erosion damage. Moreover, its well-developed root system also enhances the ability of the slope soil to resist erosion and plays a major role in slope stabilization. Therefore, in the experiment, although the surface runoff observed in the second stage on the *Zoysia japonica* slope accounts for more than half of the total rainfall amount, the sediment content only reaches 0.06%. The improvement of soil erosion resistance also played a good role in stabilizing the slope. In the test, the *Zoysia japonica* slope did not slip with no obvious deformation, as shown in Fig. 3c. However, the *Nephrolepis cordifolia* slope had a certain degree of slide, which was more obvious during the second rainfall, as shown in Fig. 3b. Compared to the *Zoysia japonica* slope, only the second form of rainwater was observed on the *Nephrolepis cordifolia* slope. This indicates that *Nephrolepis cordifolia* exerts a major effect on restraining slope surface runoff. The aboveground stem leaf of *Nephrolepis cordifolia* is abundant, with tufted leaves, i.e., folium simplicipinnatum, densely arranged in a tile-like shape, which are 30–70 cm long and 3–5 cm wide, so rainwater is intercepted multiple times, the shape and size of raindrops are altered, and raindrop energy is consumed. This effect is manifested in the overall inhibition of rainwater infiltration at the beginning of rainfall. However, this also reduces the probability of direct rainfall impact on the slope, which decreases the sealing and water film effects caused by splash erosion and increases the contact range between raindrops and soil. This contributes to rainwater infiltration during the later period, which is verified by the fact that no surface runoff occurs during the *Nephrolepis cordifolia* slope model test.

According to whether the water seepage out of the slope, the second type of infiltration rainfall can be divided into water storage part and seepage part. During the two rainfall processes, the water storage capacity of the exposed soil slope was almost unchanged, while that of the *Zoysia japonica* slope was slightly higher than that of the former with a slight increase. The water storage capacity of the *Nephrolepis cordifolia* slope in the first stage was more than four times that of the former two slopes, but it decreased sharply in the second stage. Lu et al.^68^ pointed out that the water content of saturated soil generally tends to increase with the increase of vegetation amount, and the range can be up to 34%. Plant roots can cause compaction, particle redirection and macropore development of the soil at a certain scale, which can lead to the increase of water content of the saturated soil. Bormann et al. (2007) also showed that the increase of saturated soil water content caused by roots could reach 19%, 16% and 15% for trees/shrubs, grasses and crops, respectively. In this experiment, the vegetation amount above the surface of *Zoysia japonica* slope was less, and the root system was shallow, and most of the thickness the slope soil layer was still similar to that of the exposed soil slope, so the water storage capacity of the *Zoysia japonica* slope in the first stage was only slightly greater than that of the exposed one. For the *Nephrolepis cordifolia*, the vegetation amount above the surface is much larger than that of *Zoysia japonica*, the interception effect of stem and leaf is more obvious. The root system of *Nephrolepis cordifolia* is largely composed of creeping main stem, which is unbranched, sparsely covered with scales, with slender brown fibrous^77^, Therefore, it has a much lower degree of compaction to the shallow soil layer than that of *Zoysia japonica*, with a weak blocking effect on rainwater infiltration, which was the reason why the lag degree of rainwater infiltration at different depths of the *Nephrolepis cordifolia* slope is basically the same in the model test. However, the taproots of *Nephrolepis cordifolia* can reach 30 cm into the deep soil and have tubers (as shown in Fig. 10b), the water content of saturated soil can be greatly increased in a larger depth range^68^. Affected by the above two aspects, the *Nephrolepis cordifolia* slope had much more water storage capacity than that of exposed slope and *Zoysia japonica* slope in the first stage, corresponding to the soil saturation was also much higher than the latter two, which directly contributed to the difficult of *Nephrolepis cordifolia* slope soil further absorb rainwater, the seepage of the most infiltrated rainwater, and only 2.4% of the second rainfall was stored in the slope at last. Nevertheless, for the whole process, the total water storage capacity of the *Nephrolepis cordifolia* slope was 21.1% of the total rainfall, while that of the exposed and the *Zoysia japonica* slopes were 8.8% and 10.4%, respectively, The exposed slope had minimal storage capacity, this was corresponding to the studies of Bormann et al. (2007) and Lu et al.^68^.

Due to the differences of the water storage capacity in the first stage, the rainwater seepage of the exposed slope was the largest, followed by that of the *Zoysia japonica* slope, and the *Nephrolepis cordifolia* slope had the minimum seepage. In addition, the start time of rainwater infiltration of vegetation slope was earlier than that of exposed slope owing to the existence of root system. Under the combined influences of these two aspects, in the first stage, the equivalent permeability coefficient of the exposed slope was the largest, while that of the *Nephrolepis cordifolia* slope is the minimum. However, in the first stage, the daubing and water film effects were generated on the surface of the exposed slope through raindrop splash erosion, and the water adsorption capacity of soil decreased significantly due to the reduce of matric suction for the increase of soil saturation, and the compaction of rainwater seepage leaded to narrow or even the blockage of seepage channels in soil. Therefore, it was more difficult for rainwater infiltration of the exposed slope in the second stage, and the total infiltration amount was less than 1/3 of that in the first stage. At the same time, the seepage of rainwater was also more difficult, and the equivalent permeability coefficient drop sharply to 24.6% of the first stage. For vegetation slopes, the impact energy of rainwater was mostly absorbed by vegetation stem leaf, and the effects of daubing and water film were almost non-existent. The root system of *Zoysia japonica* was very shallow, the root soil interface was only benefit for seepage in a very thin soil layer of the slope, and the seepage in most soil layers was the same as in the exposed slope, so the influence of the third side is still prevalent and plays a leading role. Therefore, in addition to the root soil interface in the shallow layer, most of the seepage is still carried out along the pores in the soil, so the influence of the compaction effect of rainwater seepage was still prevalent and played a leading role. Therefore, compared with the exposed slope, the decrease of seepage coefficient of *Zoysia japonica* slope was relatively small, which was about 50% of the first stage. However, the seepage channel in the soil had better connectivity due to the increase of soil saturation, so the seepage time was earlier in the second stage. Compared with *Zoysia japonica*, the root system of *Nephrolepis cordifolia* is thicker and penetrates deeper into the soil, it is a good seepage channel at root-soil interface^78^. The increase of saturated soil water content caused by roots itself also means the increase of soil pore ratio, so the larger of the area of rainwater seepage channel (Bormann et al. 2007)^68^. The favorable effects of these two aspects made the rainwater can all infiltrate into the *Nephrolepis cordifolia* slope in the second stage, and there was no surface runoff, so the equivalent permeability coefficient increased by 67% instead of decreasing.

Traffic engineering construction will significantly change the local topography, destroy the surface vegetation, induce scouring, erosion and other hydrological damage, resulting in the loss of soil and water, while the increase of soil loss rate will reduce soil fertility^79^, and further affect the growth of surface vegetation, forming a vicious circle. Therefore, according to the above analysis, the following implications can be obtained for the traffic engineering construction in the granite residual land area. First, the impact, splash erosion and seepage compaction of rainfall can significantly change the hydrological effect of the exposed slope, and which can further greatly increase the erosion and soil and water loss if there has a secondary heavy rainfall. Second, vegetation protection can effectively regulate the hydrological effect of slope and enhance the self-protection ability of slope in response to continuous heavy rainfall, but the regulation mechanism and ways of different vegetation types are different. The stem leaf of *Zoysia japonica* has the role of multiple blocks, which can effectively reduce the flow velocity of surface runoff and reduce scouring, and play a leading role in regulating the hydrological effect of slope. Roots of *Zoysia japonica* can reduce the infiltration of rainwater and increase the surface runoff to a certain extent, but have little influence on the infiltration characteristics of slope soil. However, the developed shallow root system also strengthens the anti-erosion ability of slope soil and plays a good effect of slope protection. Both the stem leaf and the root system of *Nephrolepis cordifolia* have an important influence on the hydrological effect of the slope. The large amount of stem leaf can effectively absorb rainwater energy, avoid the direct impact, splash erosion of soil, and let rainwater stable infiltration, which can successfully restrain the surface runoff under continuous heavy rainfall. The root system greatly increases the water content of saturated soil, so the water storage capacity of the *Nephrolepis cordifolia* slope is much higher than that of the exposed and *Zoysia japonica* slopes. Therefore, for the residual soil slope without stability problem, it can be considered to plant *Nephrolepis cordifolia* to conserve soil and water. However, for slopes with potential stability risks, it should be used with caution, because the strong water storage capacity will greatly increase the soil weight and slope sliding force, and high water content will normally reduce the soil strength, which is not conducive to slope stability, especially in the case of heavy rainfall after drought, this effect will be more significant.

## Conclusion

The granite residual soil area in Guangdong region of China is often subjected to heavy rainfall caused by two typhoons landing or two typhoons landing continuously. In order to study the hydrological effects of vegetation granite residual soil slopes under secondary heavy rainfall, model tests of three planted slopes were carried out under artificial rainfall. The variation in slope surface runoff, soil water content and rainwater discharge over time during two rainfall events was measured, and the following conclusions were obtained after result analysis:

(1) Heavy rainfall can significantly change the hydrological effect of the exposed slope. If the exposed slope suffers a second heavy rainfall, the degree of slope scouring and soil erosion damage will increase greatly than in the first rainfall.

(2) Different vegetation species have different mechanisms and ways to regulate the hydrological effect of slope, and the multiple hindrances of the stem leaf of *Zoysia japonica* plays a leading role in regulating the hydrological effect of slope. Although the root system has little effect on the permeability and water storage capacity of slope soil, it improves the erosion resistance of it.

(3) Both the stem leaf and root system of *Nephrolepis cordifolia* have important roles on the hydrological effect. The stem leaf can stabilize the infiltration of rainwater, and successfully inhibit the surface runoff under secondary continuous heavy rainfall. The root system significantly enhances the water storage capacity of the slope, and greatly increases the permeability of the slope soil in the second rainfall, which is totally different from that of the exposed and *Zoysia japonica* slopes.

(4) Zoysia is a suitable vegetation species in terms of slope protection because of its comprehensive slope protection effect. *Nephrolepis cordifolia* should be cautiously planted as slope protection vegetation. Only on slopes with no stability issues should *Nephrolepis cordifolia* be considered to preserve soil and water.
